# Heterogeneous Nuclear Ribonucleoprotein A1 Loads Batched Tumor-Promoting MicroRNAs Into Small Extracellular Vesicles With the Assist of Caveolin-1 in A549 Cells

**DOI:** 10.3389/fcell.2021.687912

**Published:** 2021-06-17

**Authors:** Yangyang Li, Jian Zhang, Sha Li, Chongye Guo, Qian Li, Xin Zhang, Meng Li, Shuangli Mi

**Affiliations:** ^1^Key Laboratory of Genomic and Precision Medicine, Beijing Institute of Genomics, Chinese Academy of Sciences, China National Center for Bioinformation, Beijing, China; ^2^University of Chinese Academy of Sciences, Beijing, China

**Keywords:** miRNA—microRNA, SUMOylation, extracellular vesicles, RNA binding protein, sorting mechanism

## Abstract

MicroRNAs in small extracellular vesicle (sEV-miRNAs) have been widely investigated as crucial regulated molecules secreted by tumor cells to communicate with surroundings. It is of great significance to explore the loading mechanism of sEV-miRNAs by tumor cells. Here, we comprehensively illustrated a reasoned loading pathway of batched tumor-promoting sEV-miRNAs in non-small cell lung cancer (NSCLC) cell line A549 with the application of a multi-omics method. The protein heterogeneous nuclear ribonucleoprotein A1 (hnRNPA1) was strictly selected as a powerful sEV-miRNA loading protein from miRNA-binding proteome and further verified through small RNA sequencing after hnRNPA1 silence. In terms of the mechanism, SUMOylated hnRNPA1 in sEVs was verified to control sEV-miRNA loading. Subsequently, as a scaffolding component of caveolae, caveolin-1 (CAV1) was detailedly demonstrated to assist the loading of SUMOylated hnRNPA1 and its binding miRNAs into sEVs. Inhibition of CAV1 significantly prevented SUMOylated hnRNPA1 from encapsulating into sEVs, resulting in less enrichment of sEV-miRNAs it loaded. Finally, we confirmed that hnRNPA1-loaded sEV-miRNAs could facilitate tumor proliferation and migration based on database analysis and cytological experiments. Our findings reveal a loading mechanism of batched tumor-promoting sEV-miRNAs, which may contribute to the selection of therapeutic targets for lung cancer.

## Introduction

Intercellular communication is under highly complex and rigid regulations in multicellular organisms. With the features of 30- to 150-nm diameter and abundant bioactive contents, small extracellular vesicle (sEV) has been documented as a major vehicle of intercellular communication in the past decade or so (Valadi et al., 2007). We summarized four superiorities of sEVs as intercellular information carriers. First, sEVs contain various bioactive signal cargos, including lipids, proteins, and nucleic acids (Skog et al., 2008). Second, the lipid bilayer of sEVs guarantees the stability of contents, especially the fragile single-stranded RNA, resulting in authenticity of intercellular communication (Cheng et al., 2014; Ge et al., 2014). Third, almost all bioactive cells secrete sEVs, and the alteration of its contents depends on the source and physiological status of maternal cells (Villarroya-Beltri et al., 2014; Hessvik and Llorente, 2018). For instance, tumor cells secrete more sEVs than normal cells, and different sources of cancer cells secrete different sEV-miRNA repertoires (He et al., 2014; Ogata-Kawata et al., 2014). Fourth, sEVs own some organ-targeted abilities, capable of regulating the fate of recipient cells (Hoshino et al., 2015; Zhang et al., 2015). Therefore, sEVs play significant roles in construction and balance keeping of tumor environment, intriguing us to explore the loading mechanisms of widely reported microRNAs in sEV (sEV-miRNAs).

MicroRNAs represent a class of non-coding single-stranded RNA with a feature length of 18–25 nt (Esquela-Kerscher and Slack, 2006). Tumor cells secrete a myriad of sEV-miRNAs to facilitate communication with surroundings. As we previously reviewed about sEV-miRNAs trafficking, it seems purposeful for tumor cells to secrete specific sEV-miRNAs out (Zhang et al., 2015). So far, several RNA-binding proteins (RBPs) have been announced to participate in the sorting process of sEV-miRNAs (Groot and Lee, 2020), which can be separated into the following two parts. The first part is specific-motif embedding in miRNAs, which is defined as a principle of sEV-miRNA sorting by RBPs. SUMOylated hnRNPA2B1 sorts miRNAs with a tetranucleotide motif (GGAG) into exosome (namely, sEVs) (Villarroya-Beltri et al., 2013); and SYNCRIP (hnRNPQ), serving as a component of sEV-miRNA sorting, takes part in sorting of sEV-miRNAs with another motif (GGCU) (Santangelo et al., 2016). Additionally, two other RBPs (SRSF1 and FMR1) are also involved in sEV-miRNA sorting based on the specific-motif embedding in miRNAs (Wozniak et al., 2020; Xu et al., 2020). The second part, a number of versatile RBPs [such as YBX1, Ago2, MVP, HuR, heterogeneous nuclear ribonucleoprotein A1 (hnRNPA1), lupus La protein, and EWI-2] have a positive influence on the encapsulation of one or several sEV-miRNAs (McKenzie et al., 2016; Mukherjee et al., 2016; Shurtleff et al., 2017; Teng et al., 2017; Gao et al., 2019; Temoche-Diaz et al., 2019; Fu et al., 2021). Among them, hnRNPA1 is repeatedly reported to involve in the loading of sEV-miRNA (such as miR-196a, miR-320, and miR-522) to exert biological function in different cell lines (Gao et al., 2019; Qin et al., 2019; Zhang et al., 2020), suggesting its great significance on sEV-miRNA loading. It is essential for researchers to comprehensively illustrate the dominance of hnRNPA1 in sEV-miRNA loading, for example, how many miRNA species it loads and through what mechanism it works.

The incidence rate of lung cancer is one of the highest in the world. It is very important to study the sEVs and its contents of lung cancer. However, the studies of cargo loading of sEVs in lung cancer are insufficient. In our previous study, we have reported the effect of A549 cells-derived exosomes on the protein secretome of lung fibroblasts (Zhang et al., 2021). In this study, we studied the sorting mechanism of the sEV-miRNAs in A549 cells. We identified that hnRNPA1 loaded batched tumor-promoting miRNAs into sEVs with the assist of caveolin-1 (CAV1) in A549 cells. We first identified the difference of miRNA repertoire in cells and sEVs by next-generation sequencing (NGS). Through screening the sEV-enriched miRNA (miR-320b) binding proteins identified by high-throughput mass spectrometry (MS), hnRNPA1 was regarded as the optimal candidate sEV-miRNA loading protein. Analysis of sEV-miRNAs alteration after hnRNPA1 silence further confirmed its powerful sEV-miRNA loading ability. SUMOylated hnRNPA1 was considered to be the major sEV-miRNA loading protein after detecting modification of hnRNPA1 in sEVs. Subsequently, reduction of CAV1 was found to be associated with the decrease of SUMOylated hnRNPA1 and its binding miRNAs in sEVs. Furthermore, the results of database analysis and cytology experiments exhibited that sEV-miRNAs loaded by hnRNPA1 could promote proliferation and motility of recipient cells.

## Materials and Methods

### Cell Lines

Cells were routinely incubated in a humidified chamber at 37°C with 5% CO_2_. A549 and Jurkat cells were obtained from American Type Culture Collection and grown in Roswell Park Memorial Institute (RPMI) 1640 medium supplemented with 10% fetal bovine serum (FBS) (Gibco Life Technologies, New York, United States). Human embryonic kidney 293T cells (HEK293T) were grown in Dulbecco’s Modified Eagle Medium (DMEM) supplemented with 10% FBS (Gibco Life Technologies). All media were also supplemented with 100 U/ml of penicillin and 100 μg/ml of streptomycin. All cell lines were passaged routinely after purchase, and the passage numbers were less than 30 when the experiments were performed.

### Purification and Characterization of Small Extracellular Vesicle

Complete medium was replaced by basic medium without FBS when cells were cultured about 80–90% confluence. Cell supernatants containing sEVs were collected after incubation for 48 h. The sEV pellets from cell supernatants were purified by a series of centrifugation. Briefly, cell supernatants were centrifuged at 300 *g* for 10 min to remove cells and then at 16,000 *g* for 30 min to remove cell debris. After filtration with 0.22-μm filter membrane (Millipore, Cork, Ireland), cleared supernatants were centrifuged at 120,000 *g* for 70 min using a type 70Ti rotor (Beckman Coulter, Brea, CA, United States). All centrifuge steps were kept at 4°C. The sEV pellets were resuspended in filtered phosphate-buffered saline (PBS; pH 7.4).

The particle size distribution of sEVs was measured by nanoparticle tracking analysis (NTA) using a NanoSight NS300 particle analyzer (NanoSight NTA version 2.3) following the manufacturer’s instruction (Malvern Instruments Ltd., Worcestershire, United Kingdom). Pellets of sEVs were fixed and examined using a JEM-1400 (JEOL, Tokyo, Japan) transmission electron microscopy (TEM) at an acceleration voltage of 80 kV as described (Zhang et al., 2021).

### The Location of microRNA in Cells and Small Extracellular Vesicle

PKH26 Red Fluorescent Cell Linker Mini Kit (Sigma, St. Louis, MO, United States) was used to label cell membrane according to the manufacturer’s protocol. MiR-320c with 3′-terminal labeled FAM (miR-320c-FAM) were synthesized by Sangon Biotech (Shanghai, China) and transfected into cells using Hieff Trans^TM^ Liposomal Transfection Reagent (Yeasen, Shanghai, China). Then, cells were washed three times with PBS to remove unpackaged miR-320c-FAM, and medium was replaced by the basic medium without FBS. After incubation 48 h later, sEVs derived from PKH26-labeled and miR-320c-FAM transfected cells was purified by a series of centrifugation. Cells and sEVs were analyzed by Image StreamX Imaging Flow Cytometer (Amnis Corporation, Seattle, WA, United States) according to the manufacturer’s protocol.

### Plasmid Construction and Overexpression

The full-length fragments of hnRNPA1, CAV1, CD63, and CD82 were respectively amplified from complementary DNA (cDNA) of A549 by PCR using Phusion DNA Polymerase (Thermo Fisher Scientific, Waltham, MA, United States). The fusion fragments were linked by a flexible linker (SGGGG)_3_S. HnRNPA1-eGFP was cloned into pcDNA 3.1 + using *Hin*dIII and *Xho*I endonucleases (NEB). CD82–RFP was cloned into pcDNA 3.1 + using *Eco*RI and *Xho*I endonucleases (NEB). HnRNPA1-flag, CAV1-flag, and CD63-dsRED were cloned into pLEX-MCS using *Bam*HI and *Xho*I endonucleases (NEB). All plasmids were confirmed by DNA sequencing (Sangon Biotech) before use.

To co-express the fusion fluorescent protein of hnRNPA1-eGFP and CD82–RFP, the corresponding plasmids were transfected into A549 cells using Lipofectamine-2000 (Invitrogen, Carlsbad, CA, United States) according to the manufacturer’s instruction. Fluorescent proteins were observed using confocal microscope with a 40× oil objective lens (Leica, Wetzlar, Germany). Co-localization was analyzed by ImageJ software (National Institutes of Health, Bethesda, MD, United States). Lentivirus infection was applied to generate cell lines with stable overexpression of flag-labeled proteins or CD63-dsRED. Lentivirus supernatants were generated from HEK293T packaging cells after 48 h of co-transfection of the corresponding plasmids (pLEX-MCS, p8.74, and pVSVG). Viral supernatants were added into A549 cells after filtration with a 0.45-μm filter membrane (Millipore). After infection for 48 h, puromycin was applied to screen the stable cell lines. When wild-type cells died out, the stable cell lines were kept in dose by half of puromycin for the following experiments.

### Uptake of Small Extracellular Vesicle by Cells

Labeled sEV pellets were purified from supernatants of A549 cells that overexpressed CD63-dsRED fusion protein. Then, they were incubated with A549 cells grown on a cover glass for 24 h. Subsequently, cells were washed three times with cold PBS and then fixed with 4% paraformaldehyde. After cell nuclei being labeled with DAPI, the cover glass was bonded with a glass slide. The labeled sEV pellets phagocytized by A549 were observed using confocal microscope with a 40× oil objective lens (Leica).

### Knockdown of Proteins by Short Hairpin RNAs

A549 cell line with stable knockdown of hnRNPA1, CAV1, or PTBP1 was established using pLKO.1-TRC Cloning Vector (Invitrogen) per the manufacturer’s protocol. Briefly, the short hairpin RNA (shRNA) primers targeting corresponding genes and control (CTR) oligo were respectively subcloned into lentiviral expression plasmid pLKO.1 between *Age*I and *Eco*RI sites. The constructed plasmids were confirmed by DNA sequencing (Sangon Biotech) before use. Lentivirus supernatants were generated from HEK293T packaging cells. Puromycin was applied to screen stable cell lines. The sequences of shRNA primers are listed as follows (He et al., 2007; Han et al., 2015; Liu et al., 2016):

shCTR (5′–3′): CCGGGAAGAGGACACGCCTTAGACTC TCGAGAGTCTAAGGCGTGTCCTCTTCTTTTTG
shhnRNPA1 (5′–3′): CCGGGCCACAACTGTGAAGTTAG AACTCGAGTTCTAACTTCACAGTTGTGGCTTTTTG
shPTBP1 (5′–3′): CCGGTGACAAGAGCCGTGACTAC CTCGAGGTAGTCACGGCTCTTGTCATTTTTG
shCAV1 (5′–3′): CCGGACCTTCACTGTGACGAAATC TCGAGATTTCGTCACAGTGAAGGTTTTTTG

### RNA Extraction and Quantification

Total RNA from cells was extracted using TRIzol reagent (Life Technologies, Carlsbad, CA, United States) following the manufacturer’s protocols. The quantification of intracellular RNA was detected by NanoDrop (Thermo Fisher Scientific). In the case of sEVs suspended in sterile PBS, three volumes of TRIzol LS reagent (Life Technologies) were added and incubated for 15 min prior to chloroform extraction. RNA degree glycogen (Life Technologies) was added as a carrier. After being washed with 75% alcohol two times, RNA pellets were resuspended in RNase-free water. MicroRNA Qubit kit and Qubit 3.0 (Thermo Fisher Scientific) were used to detect the concentration of sEV-RNA. For the small RNA sequencing experiment, RNA integrity was assessed using RNA Nano 6000 Assay Kit on Agilent Bioanalyzer 2100 system (Agilent Technologies, Santa Clara, CA, United States).

### Reverse Transcription and Quantity PCR

Reverse transcription of intracellular and sEV-miRNA was performed with miScript II RT kit (Qiagen, Hilden, Germany). For cDNA synthesis of sEV-miRNA, an equal amount of small RNA (based on microRNA Qubit assay) was used in every independent assay due to the absent standard internal reference. Moreover, 1 × 10^9^ copies of *Caenorhabditis elegans* miR-39-3p was added to calibrate the efficiency of RT and qPCR. For cDNA synthesis of intracellular miRNA, 50 ng of total RNA was used. RNU6 was used as internal reference. Real-time qPCR assay was performed using CFX96 Real-time PCR system (Bio-Rad, Hercules, CA, United States). The matched miScript SYBR Green PCR Kit (Qiagen) was used with the provided universal reverse primer and the miRNA specific primers (Supplementary Table 1). For detection of cell gene expression, the RT kit (Promega, Madison, WI, United States) was used for cDNA synthesis with the random primers and GAPDH was used as internal reference. Real-time qPCR was performed using CFX96 Real-time PCR system (Bio-Rad). SYBR PCR kit (KAPA Biosystems, Woburn, MA, United States) was used with gene-specific forward primers and reverse primers (Supplementary Table 1). Relative expression level was calculated by 2^–ΔΔ*CT*^ method.

### Small RNA Sequencing and Reads Mapping

Small RNA library was constructed using the NEB Next Multiplex Small RNA Library Prep Set for Illumina (Illumina, San Diego, CA, United States) following the manufacturer’s recommendations, and index codes were added to attribute sequences to each sample. PCR amplification was performed using Long Amp Taq 2 × Master Mix. PCR products were purified on an 8% polyacrylamide gel. DNA fragments corresponding to 140–160 bp (the length of small non-coding RNA plus the 3′ and 5′ adaptors) were recovered and dissolved in the elution buffer. Library quality was assessed on the Agilent Bioanalyzer 2100 system using DNA High Sensitivity Chips. Library preparations were sequenced on an Illumina HiSeq 2500 platform, and 50-bp single-end reads were generated.

The miRNA sequences were aligned against human mature miRNA downloaded from miRBase (Release 21) and non-coding RNA downloaded from GenBank^1^ (Homo_sapiens.GRCh38.ncrna) with Bowtie 2. The miRNA profiling was normalized using reads per million (RPM) mappable miRNA sequences. RPM = (number of reads mapping to miRNA/total number of reads mapping to miRNA) × 1,000,000.

### Western Blotting

Cell or sEV samples were lysed in radioimmunoprecipitation assay (RIPA) buffer. Concentrations of protein were detected using bicinchoninic acid (BCA) protein assay kit (Tiangen, Sichuan, China) according to the manufacturer’s protocol. Protein samples loaded into 10 or 12% polyacrylamide gels were separated by electrophoresis and then transferred to polyvinylidene difluoride (PVDF) membranes (Millipore). After being blocked with 5% skim milk in TBS + 0.1% Tween-20 for 2 h at room temperature, membranes were incubated overnight at 4°C with the corresponding primary antibodies: anti-hnRNPA1 (CST, Danvers, MA, United States; 1/1,000), anti-CAV-1 (ABclonal, Woburn, MA, United States; 1/1,000), anti-CD63 (Abcam, Cambridge, United Kingdom; 1/2,000), anti-TSG101 (ABclonal, 1/1,000), anti-Alix (CST, 1/1,000), anti-YBX1 (ABclonal, 1/1,000), anti-GAPDH (BioWorld, St Louis Park, MN, United States; 1/1,0000), anti-flag (Sigma, 1/3,000), anti-SUMO1 (HuaAn, Hangzhou, China; 1/1500), anti-β-actin (Sigma, 1/3,000), and anti-ENO1 (Abcam, 1/2,000). Afterward, membranes were incubated with horseradish peroxidase (HRP)-conjugated secondary antibodies for 2 h at room temperature. Three times of washing (10 min each) by Tris-buffered saline (TBS, pH 7.5) plus 0.1% Tween-20 were done before chemiluminescence detection by a luminescence imaging system (Tanon, Shanghai, China). ImageJ software was used for quantification of western blotting bands.

### MiRNA Pull-Down

About 2 × 10^7^ cells were lysed in 1 ml of lysis buffer [50 mM of Tris (pH 7.5), 150 mM of NaCl, 1 mM of EDTA, 1% of Triton X-100, and 40 U/ml of RNasin] supplemented with protease inhibitors and centrifuged at 12,000 *g*, 4°C for 10 min. Then, 0.8 ml of supernatant was precleared by incubation with streptavidin magnetic beads. MiRNAs with 3′-terminal labeled biotin and negative control miRNA oligo (5′-UUGUACUACACAAAAGUACUG-3′) were synthesized by Sangon Biotech. Streptavidin magnetic beads (50 μl) were washed three times with TBS and balanced with the equal volume of RNA capture buffer [20 mM of Tris (pH 7.5), 1 M of NaCl, and 1 mM of EDTA]. Then, 50 pmol of labeled RNA was mixed with balanced streptavidin magnetic beads for 2 h at 4°C with rotation. After streptavidin magnetic beads in conjunction with biotin-labeled miRNAs were washed three times with TBS, the precleared supernatant of cell lysis sample was added and then incubated for 4 h at 4°C with rotation. Then, beads were washed five times with TBS and resuspended in protein loading buffer. The obtained beads were boiled at 95°C for 5 min before analysis.

### Protein Identification by Mass Spectrometry

The proteins pulled down by streptavidin magnetic beads were separated by 10% sodium dodecyl sulfate–polyacrylamide gel electrophoresis (SDS-PAGE) gel. Then gel was stained using a protein silver stain kit (CWBio, Beijing, China) according to the manufacturer’s protocols. The target protein bands were excised from the gel and subjected to MS. Beijing Protein Innovation Co., Ltd. (Beijing, China) performed the MS assay.

### Flag-Labeled Protein Purification

Stable overexpression of hnRNPA1-flag or CAV1-flag cells was washed with ice-cold PBS three times before being subjected to UV crosslink (150 mJ/cm^2^). The cells were lysed in lysis buffer [50 mM of Tris (pH 7.5), 150 mM of NaCl, 1 mM of EDTA, 1% Triton X-100, and 40 U/ml of RNasin] supplemented with protease inhibitors and centrifuged at 12,000 *g*, 4°C for 10 min. ANTI-FLAG^®^ M2 Magnetic Beads (Sigma) were adopted to pull down the flag-labeled proteins. For protein analysis, the pull-down complex was boiled in protein loading buffer and analyzed by Western blotting. For miRNA analysis, the pull-down complex was digested by proteinase K, and then RNA was extracted for the subsequent RT-qPCR.

### SUMOylation Inhibitor

When cultured at about 80%–90% confluence, cells were treated with anacardic acid (AA) (50 μM) (Sigma) or dimethyl sulfoxide (DMSO) for 4 h before application of medium without FBS to collect sEVs. Supernatants were collected after 12 h, and sEV pellets were purified as described above.

### Cell Viability Assay and Wound-Healing Assay

Cell viability assay was performed using CellTiter 96^®^ AQueous Non-Radioactive Cell Proliferation Assay (MTS) (Promega) following the manufacturer’s protocols. A549 cells were incubated with 100 μg/ml of sEV pellets (Liang et al., 2016); and optical density (OD) was measured at 490 nm every 24 h and continued for 4 days. For the wound-healing assay, A549 cells were plated into a 24-well plate (1 × 10^5^ cells/well). After cell monolayers were wounded by a gap, 100 μg/ml of sEV pellets (Liang et al., 2016) was added. The gaps were observed under an inverted microscope (Leica) at specific time points (0, 24, and 48 h).

### Statistics

Results were presented as means ± SEM of at least three independent experiments. All statistical analyses were performed with Prism GraphPad 8 software. The statistical significance of two independent groups was calculated by two-tailed Student’s *t*-test. The significant difference was reflected by *p* < 0.05.

## Results

### Different microRNA Repertoire in Cells and Small Extracellular Vesicle

**FIGURE 1:**
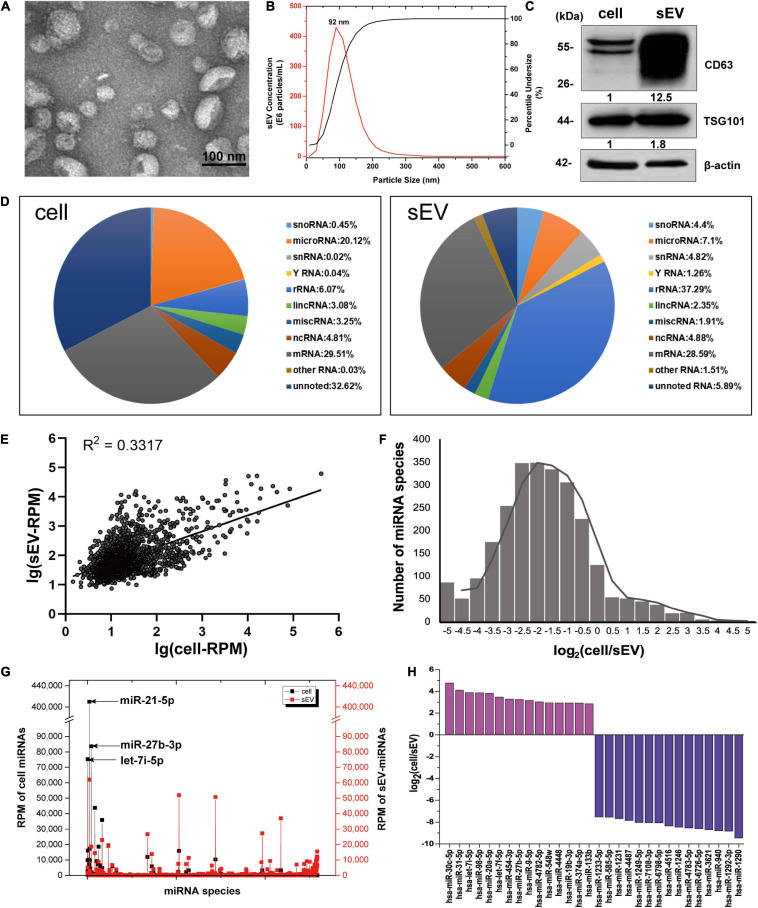
The distribution features of miRNAs in cells and sEVs. **(A)** TEM image of isolated sEV pellets. Scale bar = 100 nm. **(B)** Size distribution of sEV pellets measured by NanoSight NS300 with the peak at 92 nm. **(C)** Western blotting analysis of sEV marker proteins CD63 and TSG101. **(D)** Pie charts showing the small RNA components and percentages of cells (left) and sEVs (right) based on small RNA sequencing data. **(E)** Scatter diagram showing the expressions of miRNAs in cells and sEVs. R square of linear-regression analysis is 0.3317. **(F)** Frequency histogram showing the miRNA species enriched in sEVs (x < 0) and cells (x > 0). **(G)** RPM of miRNAs in cells and sEVs. Black line: RPM of intracellular miRNAs. Red line: RPM of sEV-miRNAs. **(H)** Histogram showing the enrichment multiple of the top 15 enriched miRNAs in cells (y > 0) and sEVs (y < 0). miRNAs, microRNAs; TEM, transmission electron microscopy; sEVs, small extracellular vesicles; RPM, reads per million.

A549-derived sEV pellets were purified through sequentially differential centrifugation steps. The cuplike morphological feature of sEVs was observed by TEM, as shown in Figure 1A. Reports from NTA revealed that the particle size distribution was about 30–150 nm with a peak at 92 nm (Figure 1B). Protein markers of sEVs (CD63 and TSG101) were routinely detected by western blotting (Figure 1C).

To verify that sEV-miRNAs come from their parental cells, a synthetic FAM-labeled miR-320c was transfected into cells; and then detection of FAM fluorescence was performed in corresponding sEVs. Cell plasma membrane and intracellular membrane system were first labeled with PKH26, resulting in red fluorescence indication of the outline of cells or sEV pellets. The labeled cells and sEV pellets derived from them were collected and subjected to Image StreamX Imaging Flow Cytometer analysis. It is obvious that FAM green fluorescence is located in the cavity of PKH26 red fluorescence region of both cells and sEVs (Supplementary Figure 1), indicating the transfer of miR-320c from cells to sEVs.

We sequenced the small RNA (<40 nt) of A549 cell and sEVs using NGS to explore their repertoires. The NGS results indicated the same RNA category but different quantities between them (Figure 1D). Regarding miRNA, it presented a weak correlation (R^2^ = 0.3317) between intracellular miRNAs and sEV-miRNAs (Figure 1E). In addition, we found several inherent distribution features of miRNAs in cells and sEVs. (1) The frequency histogram of miRNA species exhibited an extremely biased enrichment. About 90% of miRNA species [log_2_(cell/sEV) < 0] were enriched in sEVs (Figure 1F). (2) Although the majority of miRNA is relatively enriched in sEVs, several miRNAs (such as miR-21-5p, miR-27b-3p, and let-7i-5p) had quite high expression levels in cells (Figure 1G). (3) The enrichment multiple of miRNA in sEVs was larger than that in cells by analyzing the top 15 enriched miRNAs in both cells and sEVs (Figure 1H). These features fundamentally agreed with the previous results reported by Santangelo (Santangelo et al., 2016). All of these results manifest that sEV-miRNA loading is an active process, suggesting the existence of selective mechanisms for miRNA loading into sEVs.

### Heterogeneous Nuclear Ribonucleoprotein A1 Is the Optimal Candidate RNA-Binding Protein for MicroRNA in Small Extracellular Vesicle Loading

**FIGURE 2:**
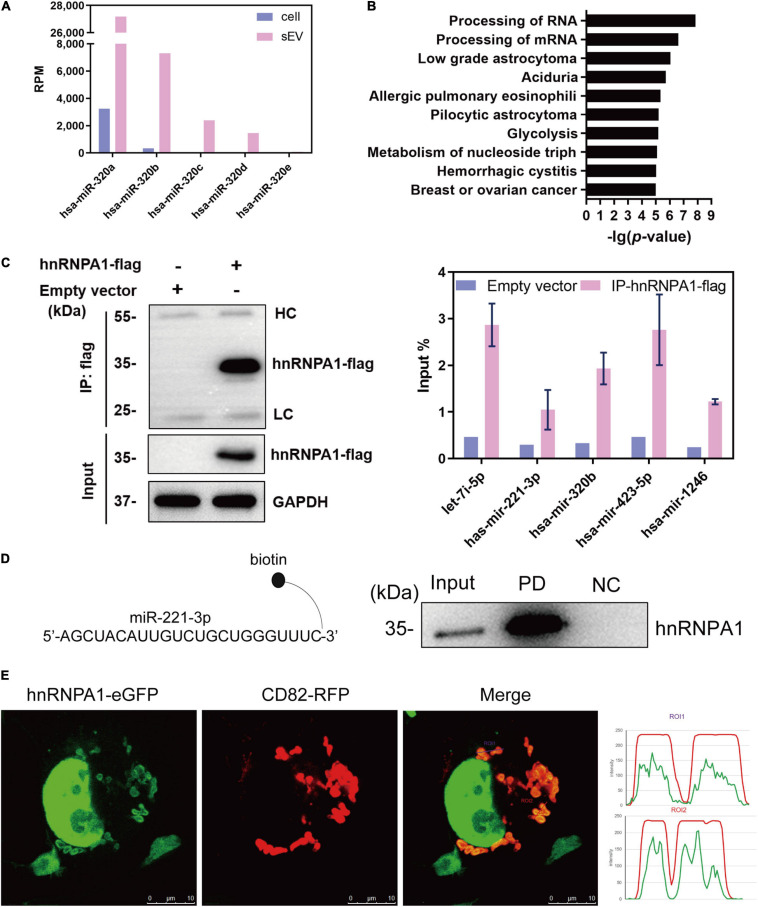
HnRNPA1 is the optimal candidate RBP for sEV-miRNA loading. **(A)** RPM of miR-320 family in cells and sEVs according to small RNA sequencing data. **(B)** Ingenuity Pathway Analysis (IPA) analysis of the 70 precipitated proteins identified by MS. Ten of the most associated pathways are shown. **(C)** Results of RNA immunoprecipitation experiments using anti-flag magnetic beads to pull down hnRNPA1-flag fusion protein, western blotting analysis of pull-down hnRNPA1-flag (left), and RT-qPCR analysis of co-precipitated miRNAs (right). HC, heavy chain. LC, light chain. Three independent experiments were performed. **(D)** Western blotting analysis of hnRNPA1 in the pull-down complex co-precipitated with biotin-labeled miR-221-3p. NC, negative control (beads + control miRNA without biotin). PD, pull-down (beads + biotin-labeled miR-221-3p). **(E)** The confocal images of RFP-labeled CD82 and eGFP-labeled hnRNPA1 in a representative A549 cell. Co-localization was analyzed in 24 cells and visualized by ImageJ software. Bars = 10 μm. hnRNPA1, heterogeneous nuclear ribonucleoprotein A1; RBP, RNA-binding protein; sEV-miRNA, microRNA in small extracellular vesicle; RPM, reads per million.

RNA-binding proteins have been documented to sort sEV-miRNAs by carrying their attached miRNAs while entering into sEVs (O’Brien et al., 2020). To precisely screen the RBPs capable of sEV-miRNA loading, sEV-enriched miRNAs binding proteins should be preferentially focused on. We selected miR-320b as a bait in consideration of its high absolute and relative quantity in sEVs according to our sequencing data (Figure 2A). Biotin-labeled miR-320b was applied to precipitate potential RBPs in the extracts of A549 cells, resulting in identification of 70 precipitated proteins by high-throughput MS (Supplementary Table 2). Function annotation revealed that most of them are associated with RNA processing (Figure 2B). We noticed hnRNP family proteins in the precipitated proteome due to their outstanding scores in MS (Table 1). Among them, HnRNPA1 had a superior abundance in cells and a satisfactory score in MS data. Furthermore, as a conserved RBP, hnRNPA1 has been reported to be involved in sEV-miRNA loading such as miR-196a, miR-320, and miR-522 (Gao et al., 2019; Qin et al., 2019; Zhang et al., 2020). Thus, we hypothesized that hnRNPA1 should be an excellent sEV-miRNA loading protein.

**TABLE 1:** HnRNP family proteins co-precipitated with miR-320b by mass spectrometry analysis.

**UniProt ID**	**Gene name**	**Protein score**	**Number of peptide**	**Number of significant peptide**	**Coverage of peptide (%)**
P31943	*HNRNPH1*	1,191	18	16	54.1
P09651	*HNRNPA1*	1,187	18	13	46.8
P55795	*HNRNPH2*	869	15	12	39.6
P52597	*HNRNPF*	828	14	12	51.1
P22626	*HNRNPA2B1*	295	14	9	46.7
P26599	*PTBP1*	191	4	3	11.5
P31942	*HNRNPH3*	167	10	7	30.9
P51991	*HNRNPA3*	54	2	2	7.1
Q13151	*HNRNPA0*	48	1	1	2.3

*hnRNP, heterogeneous nuclear ribonucleoprotein.*

The interaction of miRNAs and hnRNPA1 was verified again by performing protein and miRNA pull-down assays, respectively. HnRNPA1-flag fusion protein was overexpressed using a lentivirus infection system and then pulled down with the application of anti-flag magnetic beads (Figure 2C, left). According to sEV-miRNA sequencing data, five highly expressed miRNAs in sEVs with RPM larger than 10,000 were randomly selected as representative detection objects in the pull-down complex of hnRNPA1-flag (Figure 2C, right). Conversely, we labeled miR-221-3p, which had a weaker combination with hnRNPA1 in these five miRNAs (Figure 2C, right), with biotin used in miRNA pull-down assay. The corresponding result of western blotting manifested the existence of hnRNPA1 in the miR-221-3p co-precipitated complex (Figure 2D). Both results confirm the tightness interaction between hnRNPA1 and the selected miRNAs.

The intracellular distribution of hnRNPA1 was explored by overexpressing hnRNPA1-eGFP fusion protein, resulting in the main nucleus location with a small quantity interspersing in the cytoplasm (Figure 2E). When CD82–RFP fusion protein, a marker protein of multivesicular body (MVB; Gibbings et al., 2009), was co-expressed in cells, cytoplasmic hnRNPA1 was well co-localized with CD82 (Figure 2E). In general, it is thought that sEV-miRNA loading happens with the biogenesis of sEVs, especially during the invagination of MVB (Mashouri et al., 2019). Co-localization of hnRNPA1 with MVB suggests that hnRNPA1 stands a chance to be incorporated into sEVs.

### Heterogeneous Nuclear Ribonucleoprotein A1 Is a Powerful microRNA in Small Extracellular Vesicle Loading Protein

**FIGURE 3:**
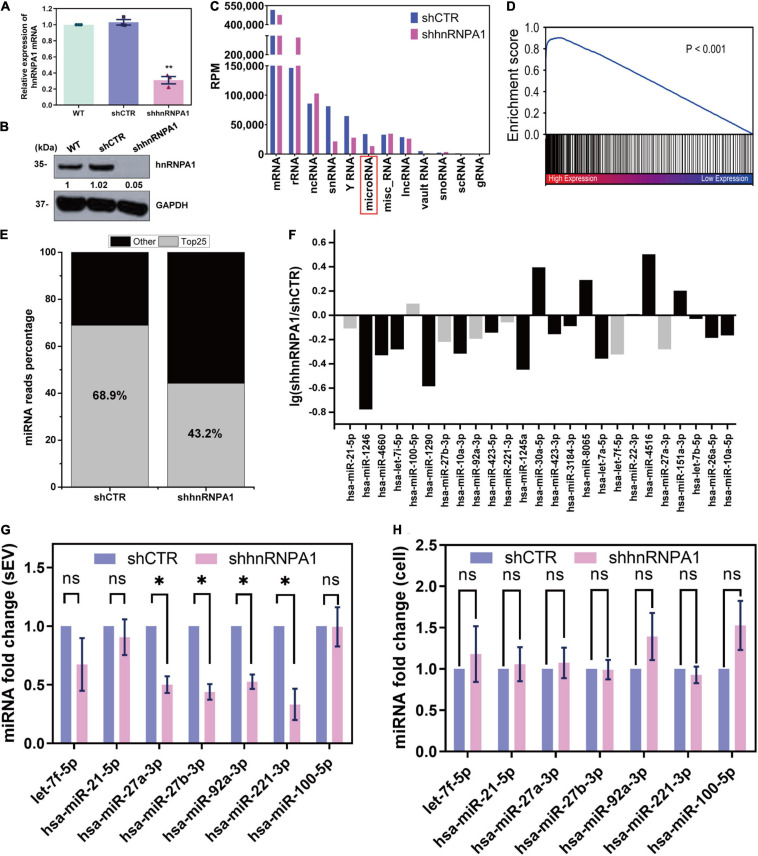
HnRNPA1 is a powerful sEV-miRNA loading protein. **(A)** RT-qPCR analysis of mRNA levels after knockdown of hnRNPA1 in A549 cells. **(B)** Western blotting analysis of protein level in shhnRNPA1 A549 cells. **(C)** RPM of different small RNA components in sEVs derived from shhnRNPA1 and control (shCTR) cells. **(D)** Enrichment analysis of the downregulated sEV-miRNAs after knockdown of hnRNPA1. X-axis: 2,585 expressed sEV-miRNA ranked by the expression level. Black lines (enriched in high expression region) represent downregulated sEV-miRNAs and white lines represent upregulated sEV-miRNAs. **(E)** Percentage of the top 25 highly expressed sEV-miRNAs derived from shCTR and shhnRNPA1 cells. Gray areas represent the top 25 miRNAs, and black areas represent other miRNAs. **(F)** Bar chart showing the alteration of top 25 highly expressed sEV-miRNAs after knockdown of hnRNPA1. MiRNAs indicated by gray bars were selected for the following RT-qPCR. RT-qPCR analysis of the relative expression levels of the selected miRNAs in shhnRNPA1 sEVs **(G)** and in shhnRNPA1 cells **(H)**. The significant differences were reported for three independent experiments (ns, no significance; ^∗^*p* < 0.05; ^∗∗^*p* < 0.01, two-tailed *t*-test). hnRNPA1, heterogeneous nuclear ribonucleoprotein A1; sEV-miRNA, microRNA in small extracellular vesicle; RPM, reads per million.

In order to investigate whether hnRNPA1 alters the profiling of sEV-miRNAs, we knocked down hnRNPA1 by shRNA (Figures 3A,B), and then we detected the variations of small RNAs in sEVs by NGS. The length distributions of total sEV RNA seemed to have no difference based on the results of Agilent Bioanalyzer 2100 System (Supplementary Figure 2). Several small RNA categories including miRNA were obviously decreased after knockdown of hnRNPA1 when compared with control (Figure 3C). In regard to the variation of miRNA profiling, we found that (1) most downregulated miRNAs were highly expressed sEV-miRNAs (Figure 3D) and (2) the top 25 highly expressed miRNAs occupied 68.9% of total miRNAs in control sEVs, dropping to 43.2% in hnRNPA1-suppressive sEVs (Figure 3E). Furthermore, most of these miRNAs (19/25) also expectedly showed a decreased level in hnRNPA1-suppressive sEVs (Figure 3F), indicating a powerful sEV-miRNA loading ability of hnRNPA1 in A549 cells.

Then we randomly selected several miRNAs from Figure 3F to perform RT-qPCR assays. MiR-100-5p loading into sEVs may not be associated with hnRNPA1 because its quantity was not changed significantly after hnRNPA1 knockdown according to the sequencing data. It was selected as a control to avoid experimental errors. The expression of above miRNAs decreased in hnRNPA1-suppressive sEVs, commendably consistent with our sequencing data (Figure 3G). As a control, we knocked down another heterogeneous nuclear ribonucleoprotein PTBP1 (hnRNPI; Table 1 and Supplementary Figure 3A), a conserved RBP too. However, the selected seven miRNAs were not inhibited in PTBP1-suppressive sEVs (Supplementary Figure 3B), suggesting a certain specificity of hnRNPA1 in loading sEV-miRNAs. Given that hnRNPA1 regulates biogenesis of some miRNAs (Guil and Caceres, 2007; Michlewski and Caceres, 2010), it is possible that a decrease of sEV-miRNAs may ascribe to the disturbance of miRNAs biogenesis in cells after knockdown of hnRNPA1. We detected these miRNAs without alteration in A549 cells after knockdown of hnRNPA1 (Figure 3H), thus excluding this possibility.

### SUMOylated Heterogeneous Nuclear Ribonucleoprotein A1 Controls microRNA in Small Extracellular Vesicle Loading

**FIGURE 4:**
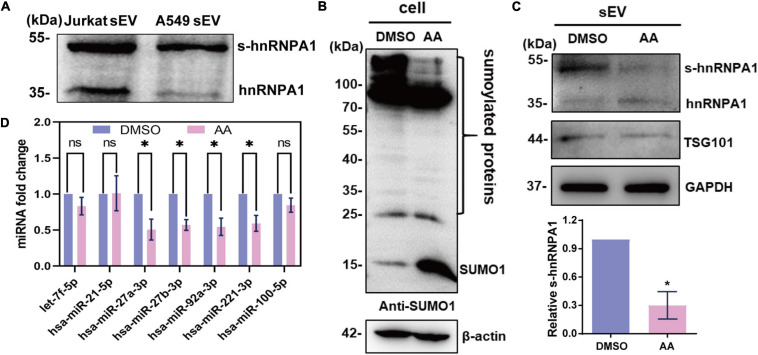
SUMOylated hnRNPA1 controls sEV-miRNA loading. **(A)** Western blotting showing the expression of hnRNPA1 including SUMOylated hnRNPA1 in sEVs derived from Jurkat and A549 cells. **(B)** The protein SUMOylation level in cells treated with DMSO and AA. Anti-SUMO1 small molecule antibody was used for western blotting. **(C)** Corresponding SUMOylated hnRNPA1 in sEVs derived from cells treated with DMSO and AA (top panel). Statistical analysis showing the ratio of SUMOylated hnRNPA1 and hnRNPA1 in three independent experiments (bottom panel). **(D)** Expression levels of selected sEV-miRNAs derived from DMSO- and AA-treated cells. The significant differences are reported for four independent experiments (ns, no significance; ^∗^*p* < 0.05, two-tailed *t*-test). hnRNPA1, heterogeneous nuclear ribonucleoprotein A1; sEV-miRNA, microRNA in small extracellular vesicle; DMSO, dimethyl sulfoxide; AA, anacardic acid.

SUMOylation has been generally found on pivotal regulatory proteins and transcription factors. This covalent modification of proteins delicately affects their functions, such as stability, subcellular localization, and interaction with other protein (Johnson, 2004). SUMOylated hnRNPA1 has been identified in cells and sEVs according to previous reports (Li et al., 2004; Villarroya-Beltri et al., 2013). As previously reported (Villarroya-Beltri et al., 2013), we identified SUMOylated hnRNPA1 in sEVs derived from Jurkat cells, and in sEVs derived from A549 cells, as expected (Figure 4A).

In order to verify the sEV-miRNA loading ability of SUMOylated hnRNPA1, the specific SUMOylation inhibitor AA was applied to treat A549 cells (Fukuda et al., 2009). SUMO1 small molecule lost its conjunct ability with proteins, resulting in the increase of free SUMO1 molecules, while all the SUMOylated proteins (especially the SUMOylated proteins lager than 100 kDa) were obviously decreased in AA-treated cells (Figure 4B). Consequently, the expression level of SUMOylated hnRNPA1 was also expectedly decreased in sEVs (Figure 4C). When variations of sEV-miRNA were verified, their expression pattern was similar with sEV-miRNA expression pattern after knockdown of hnRNPA1 (Figure 4D). From these results, we can conclude that SUMOylated hnRNPA1 controls sEV-miRNA loading.

### Caveolin-1 Assists SUMOylated Heterogeneous Nuclear Ribonucleoprotein A1 Loading microRNA in Small Extracellular Vesicle

**FIGURE 5:**
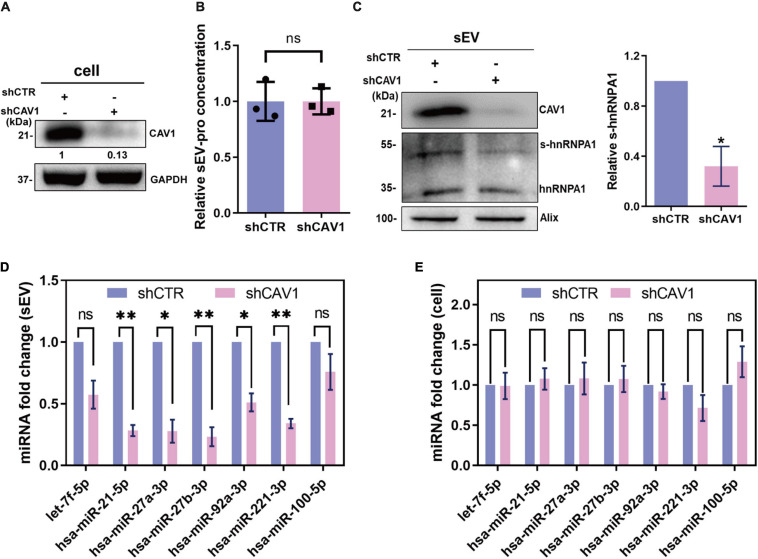
CAV1 assists SUMOylated hnRNPA1 loading sEV-miRNA. **(A)** CAV1 protein level in shCAV1 cells. **(B)** Protein concentration of sEVs derived from shCAV1 and control cells. **(C)** CAV1 and hnRNPA1 protein levels in sEVs derived from shCAV1 cells (left). Statistical analysis showing the ratio of SUMOylated hnRNPA1 and hnRNPA1 in three independent assays (right). Relative expression level of the selected miRNAs in sEVs **(D)** and shCAV1 cells **(E)**. Statistically significant differences are reported for three independent experiments (ns, no significance; ^∗^*p* < 0.05; ^∗∗^*p* < 0.01, two-tailed *t*-test). CAV1, caveolin-1; hnRNPA1, heterogeneous nuclear ribonucleoprotein A1; sEV-miRNA, microRNA in small extracellular vesicle.

Given that CAV1 regulates protein components of extracellular vesicles without altering the sEV secretion (Campos et al., 2018), it is of great interest to explore whether CAV1 has effects on sEV-miRNA loading by SUMOylated hnRNPA1. To this end, we knocked down CAV1 by shRNA in A549 cells (Figure 5A), and we detected the expression level of hnRNPA1 in sEVs. That the secretion amount of sEVs has no difference between shCAV1 and shCTR cells was demonstrated by detecting the concentration of sEV protein (Figure 5B). The western blotting results showed that SUMOylated hnRNPA1 apparently decreased in sEVs after knockdown of CAV1 (Figure 5C).

As regards the expression alteration of miRNAs, the miRNAs loaded by SUMOylated hnRNPA1 were not obviously altered in cells while significantly repressed in sEVs after knockdown of CAV1 (Figures 5D,E). Furthermore, these miRNAs were more repressed in CAV1-deprived sEVs when compared with hnRNPA1-deprived sEVs (Figures 3G, 5E). One of the possibilities is that CAV1 itself binds miRNAs and loads them into sEVs, resulting in the more repressed sEV-miRNAs. To examine the miRNA binding ability of CAV1, we purified CAV1-flag fusion protein from overexpressed cells and detected their attached miRNAs. However, a negative result was obtained, indicating the lack of miRNA binding ability of CAV1 (Supplementary Figure 4A). With the result of scarce miR-221-3p binding in CAV1 (Supplementary Figure 4B), we concluded that there was no interaction between CAV1 and miRNAs. Therefore, we speculated that this phenomenon might result from that CAV1 mediating other RBPs loading into sEVs. Actually, we indeed detected many other proteins, including YBX1, a reported sEV-miRNA sorting RBP, that were depressed in CAV1-deprived sEVs (Supplementary Figure 4C).

### MicroRNAs in Small Extracellular Vesicle Loaded by Heterogeneous Nuclear Ribonucleoprotein A1 Facilitate Tumor Proliferation and Migration

**FIGURE 6:**
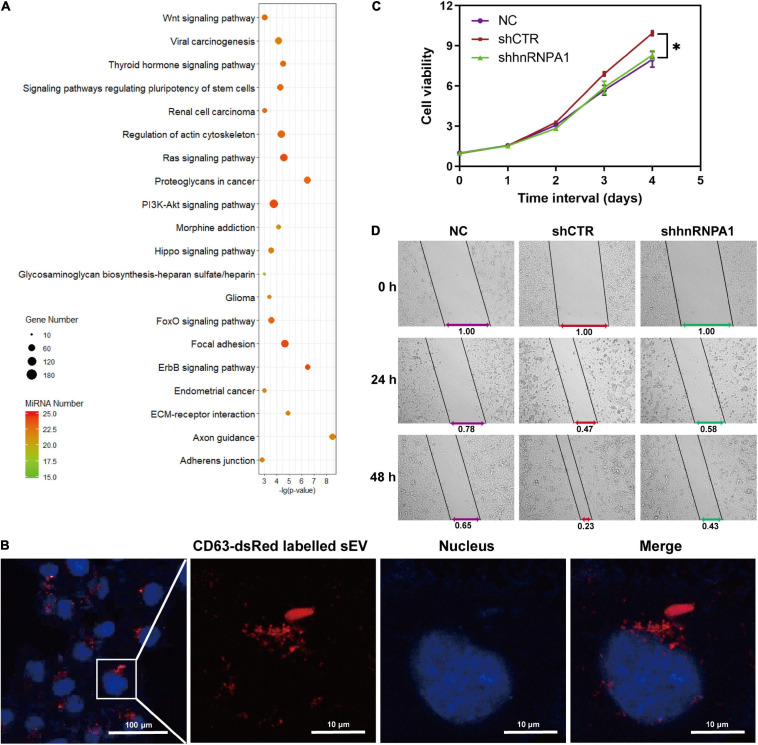
sEV-miRNAs loaded by hnRNPA1 facilitate tumor proliferation and migration. **(A)** Bubble chart showing the 19 downregulated sEV-miRNA-associated KEGG pathways. DIANA TOOLS-mirPath v.3 was used, and 20 of the most associated pathways are shown. **(B)** Confocal microscope images showing CD63-dsRed-labeled sEVs uptaken by A549 cells (sEVs, red; and nucleus, blue). **(C)** Cell viability of A549 (*p < 0.05, error bars represent SEM). **(D)** Wound-healing assay of A549 cells treated with different sEVs. NC, A549 cells without exogenous sEVs; shCTR, A549 cells adding sEVs derived from shCTR cells; shhnRNPA1, A549 cells adding sEVs derived from shhnRNPA1 cells. sEV-miRNA, microRNA in small extracellular vesicle; hnRNPA1, heterogeneous nuclear ribonucleoprotein A1; KEGG, Kyoto Encyclopedia of Genes and Genomes.

Tumor cells derived from sEV-miRNAs modify tumor microenvironment continually and contribute to tumor progression (Cereghetti and Lee, 2014; Singh et al., 2014). We aimed to explore the cancer-associated function of whole sEV-miRNAs that were loaded by hnRNPA1 integrally. As mentioned above, most of the sEV-miRNAs (19/25) in Figure 3F were downregulated after knockdown of hnRNPA1 according to our sequencing data. Therefore, we first subjected these total 19 downregulated miRNAs to Kyoto Encyclopedia of Genes and Genomes (KEGG) pathway analysis (Vlachos et al., 2015), resulting in several significant cancer-associated pathways, such as extracellular matrix (ECM)–receptor interaction, Wnt signaling pathway, and PI3K–Akt signaling pathway (Figure 6A). After sEV uptake was verified by observing the immunofluorescence labeled with sEVs in recipient cells (Figure 6B), A549 cells were incubated with sEV pellets derived from shhnRNPA1 cells (shhnRNPA1-sEV group) and control sEV pellets (shCTR-sEV group). An increasing proliferation of recipient cells was recorded in the shCTR-sEV group when compared with the normal control (NC) A549 cells without exogenous sEVs. The proliferation of A549 cells in the shhnRNPA1-sEV group returned to the level of the NC group (Figure 6C). Similarly, the result of wound-healing assay also showed an increased migration in the shCTR-sEV group and an inhibited migration in the shhnRNPA1-sEV group (Figure 6D). According to these results, we thought that this phenomenon in which the proliferation and migration of A549 cells could not be promoted by adding shhnRNPA1-sEV was due to the deficiency of intercellular communication, which is transferred through sEV-miRNAs loaded by hnRNPA1. That is to say, sEV-miRNAs loaded by hnRNPA1 promote tumor proliferation and migration.

## Discussion

In this report, we delineated that hnRNPA1 loaded batched sEV-miRNAs, with CAV1 serving as an assistant. Moreover, sEV-miRNAs loaded by hnRNPA1 facilitated tumor proliferation and migration. Although hnRNPA1 is mainly located in the nucleus, a novel nuclear localization signal with a 38 amino acid domain also enables nuclear export of hnRNPA1 (Michael et al., 1995). In our results, we observed a small quantity of hnRNPA1 interspersing in the cytoplasm and co-localized with MVB. With the highly conserved RBP ability, hnRNPA1 directly interacts with diverse RNAs including miRNA and participates in several RNA processes, such as mRNA transport, alternative splicing, and metabolism (Roy et al., 2017). The extremely complicated sEV-miRNA sorting needs kinds of mechanisms to collaborate. Although we illuminated the powerful sEV-miRNA loading potential of hnRNPA1 in A549 cells, we must confess on the existence of other RBPs or pathways to load sEV-miRNAs for the following two reasons. First, several miRNAs among the top 25 highly expressed sEV-miRNAs were indeed not decreased after knockdown of hnRNPA1, although most of them had a declining level. Second, let-7f-5p and miR-21-5p only showed a decreasing trend without significant difference in the RT-qPCR results.

A series of RBPs have been reported to be involved in sEV-miRNA sorting, and some of them sorted miRNAs with specific motifs. However, we did not observe obvious motifs in miRNAs loaded by hnRNPA1, which might be ascribed to its huge binding amount of RNA. SUMOylation is deemed as a mechanism of hnRNPA2B1 entering into exosomes due to more enrichment of SUMOylated hnRNPA2B1 in exosomes (Villarroya-Beltri et al., 2013). In our study, we verified SUMOylated hnRNPA1 in sEVs derived from Jurkat cells (Villarroya-Beltri et al., 2013) and in sEVs derived from A549 cells, suggesting the wide existence of SUMOylated hnRNPA1 in sEVs. With the treatment of AA inhibitor, SUMOylated hnRNPA1 and sEV-miRNAs loaded by SUMOylated hnRNPA1 both had a certain reduction, indicating that SUMOylated hnRNPA1 partially controlled sEV-miRNA loading.

Loading of sEV contents happens during the biogenesis of sEVs, especially during the invagination of MVBs (Mashouri et al., 2019). CAV1 not only is the scaffolding protein of caveolae but is also involved in other critical cell processes such as proliferation, apoptosis, and differentiation (Jin et al., 2011). CAV1 spreads over several organelles in the cytoplasm such as Golgi apparatus, endoplasmic reticulum, early endosome, late endosome/MVB, and lysosome and participates in protein trafficking (Jin et al., 2011; Chen et al., 2013). In high metastatic breast cancer cells, CAV1 mediates the loading of adhesion-related proteins into EVs, promoting migration and invasion of recipient cells (Campos et al., 2018). In addition, CAV1 has been reported to play roles in cargo sorting of different EV subtypes. For example, with oxidative stress induction of CAV1 tyrosine-14 phosphorylation, CAV1 mediates the encapsulation of hnRNPA2B1 and its binding miRNAs into microvesicles, a kind of EVs shedding from cell plasma membrane (Lee et al., 2019). In our results, CAV1 facilitated SUMOylated hnRNPA1 to load into sEVs, along with sEV-miRNAs loaded by SUMOylated hnRNPA1. Furthermore, we obviously observed the alteration of other protein cargos in sEVs including sEV-miRNA sorting protein YBX1 after knockdown of CAV1 (Shurtleff et al., 2017). The mechanisms of CAV1 loading sEV cargos need further exploration.

Tumor-derived sEV-miRNAs have been proposed to contribute to several cancer-associated progressions including proliferation, immune escape, tumor microenvironment maintenance, and metastasis (Tominaga et al., 2015; Fanini and Fabbri, 2017). In our data, sEV-miRNAs loaded by hnRNPA1 significantly promoted tumor proliferation and migration *in vitro*, indicating their status of tumor “accomplices.” Thus, it is reasonable to conclude that investigation of sEV-miRNA loading mechanism is of great importance to learn tumor cells, allowing a new optional therapeutic strategy for cancers.

Extracellular vesicles have been applied to deliver drugs including small interfering RNA (siRNA) for the therapy of cancers and neurologic diseases (Shahabipour et al., 2017). Encapsulation of synthetic siRNA into sEVs is one of the technically pivotal steps to realize sEV delivery. Traditional methods including electroporation and transfection-based approaches are usually somewhat unsatisfactory. For example, electroporation shows that inefficient and transfection-based approaches bring undesirable toxicity and side effects (Shtam et al., 2013). Genetic modification has been used to optimize the target ability of sEVs (Alvarez-Erviti et al., 2011), allowing us to apply this technology to encapsulate siRNAs into sEVs *in vitro* based on RBP-associated sEV-miRNA loading to promote the application of innovative drug delivery by sEVs.

In conclusion, our findings revealed powerful sEV-miRNA loading ability of hnRNPA1, which mediates the encapsulation of batched tumor-promoting sEV-miRNAs. In addition, CAV1 assisted the loading of SUMOylated hnRNPA1 (majority modification form of hnRNPA1 in sEVs) and its binding miRNAs into sEVs.

## Data Availability Statement

The raw sequence data reported in this project have been deposited in the Genome Sequence Archive in BIG Data Center, Beijing Institute of Genomics (BIG), Chinese Academy of Sciences, under accession number of CRA001469 that are publicly accessible at http://bigd.big.ac.cn/gsa. Further inquiries can be directed to the corresponding author.

## Author Contributions

SM conceived and directed the study. SM and YL designed the experiments. YL performed the experiments and statistical analyses. SL, ML, and XZ performed the experiments. JZ, CG, and QL performed the sequencing and bioinformatic analyses. YL, ML, and SM wrote the manuscript. All authors discussed the results and approved the manuscript.

## Conflict of Interest

The authors declare that the research was conducted in the absence of any commercial or financial relationships that could be construed as a potential conflict of interest.

## Footnotes

1 http://www.ncbi.nlm.nih.gov/
