# Supplementary material for: Heterogeneous Nuclear Ribonucleoprotein A1 Loads Batched Tumor-Promoting MicroRNAs Into Small Extracellular Vesicles With the Assist of Caveolin-1 in A549 Cells
Source: Front Cell Dev Biol. 2021 Jun 17;9:687912. doi: 10.3389/fcell.2021.687912 (PMC8245771; doi:10.3389/fcell.2021.687912)
Supplement: Supplementary file 1 [file Data_Sheet_1.docx]

Supplementary Material

Supplementary figures


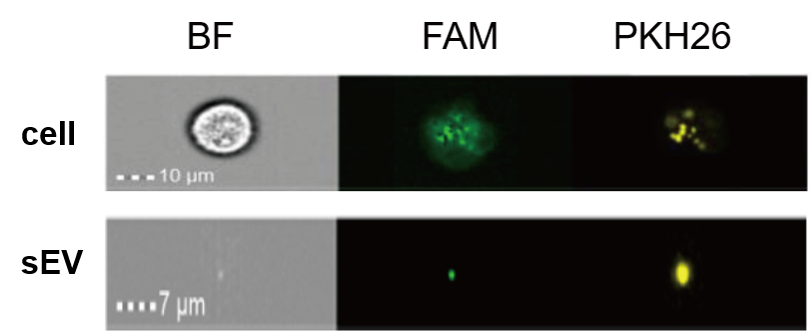


**Supplementary Figure 1.** Distribution of FAM-labeled miR-320c in single cell and in single sEV. BF, bright field.


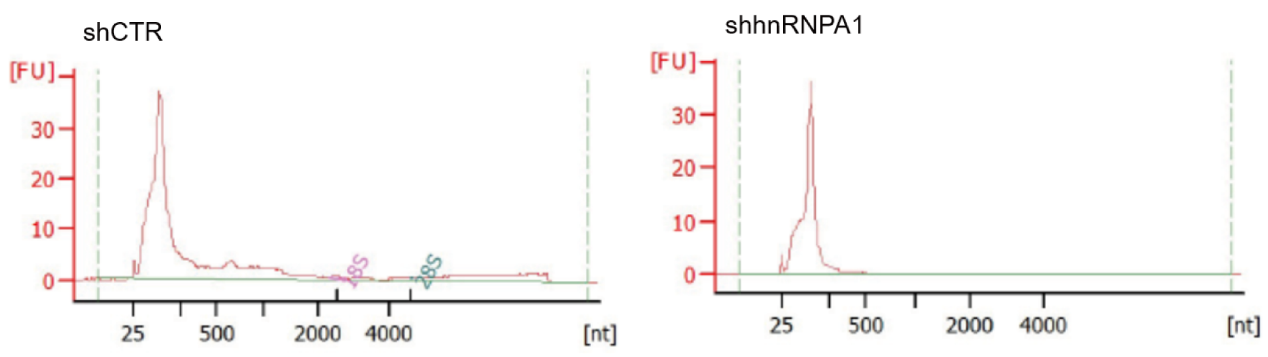


**Supplementary Figure 2.** Length distributions of sEV total RNA derived from shCTR (left) and shhnRNPA1 (right) cells analyzed by Agilent Bioanalyzer 2100 system.


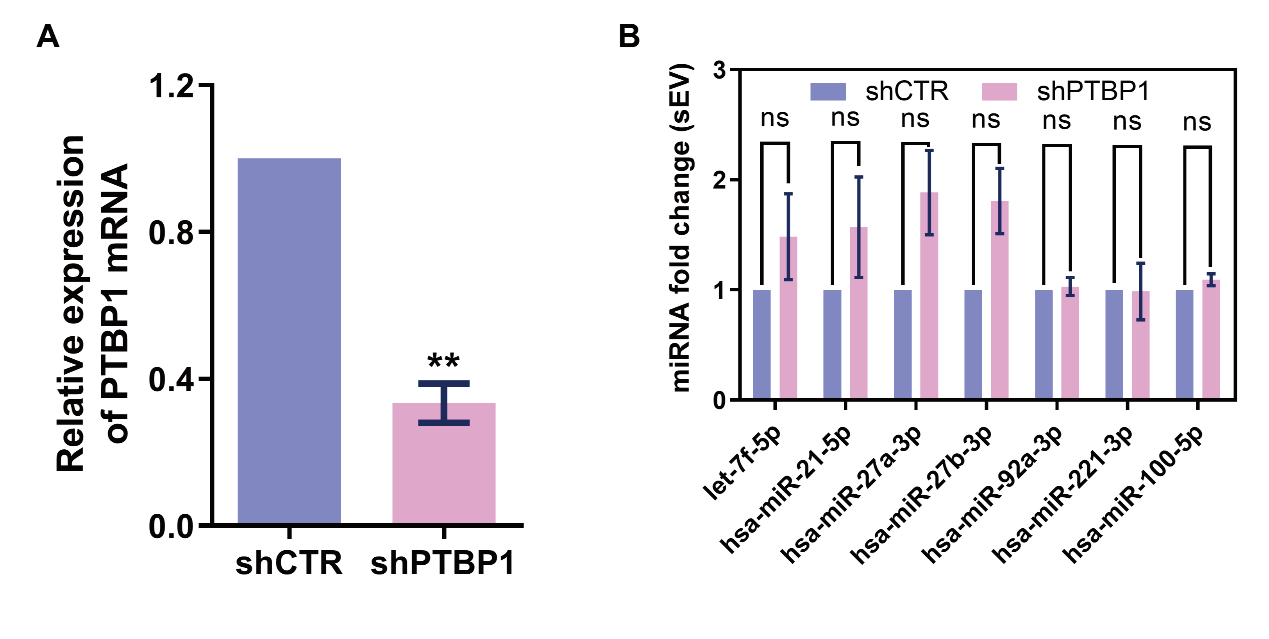


**Supplementary Figure 3.** RT-qPCR analysis of PTBP1 mRNA level in shPTBP1 cells (A) and the selected miRNAs in sEV after knockdown of PTBP1 (B). Statistically significant differences were reported for three independent experiments. (**p < 0.01, two-tailed *t*-test).


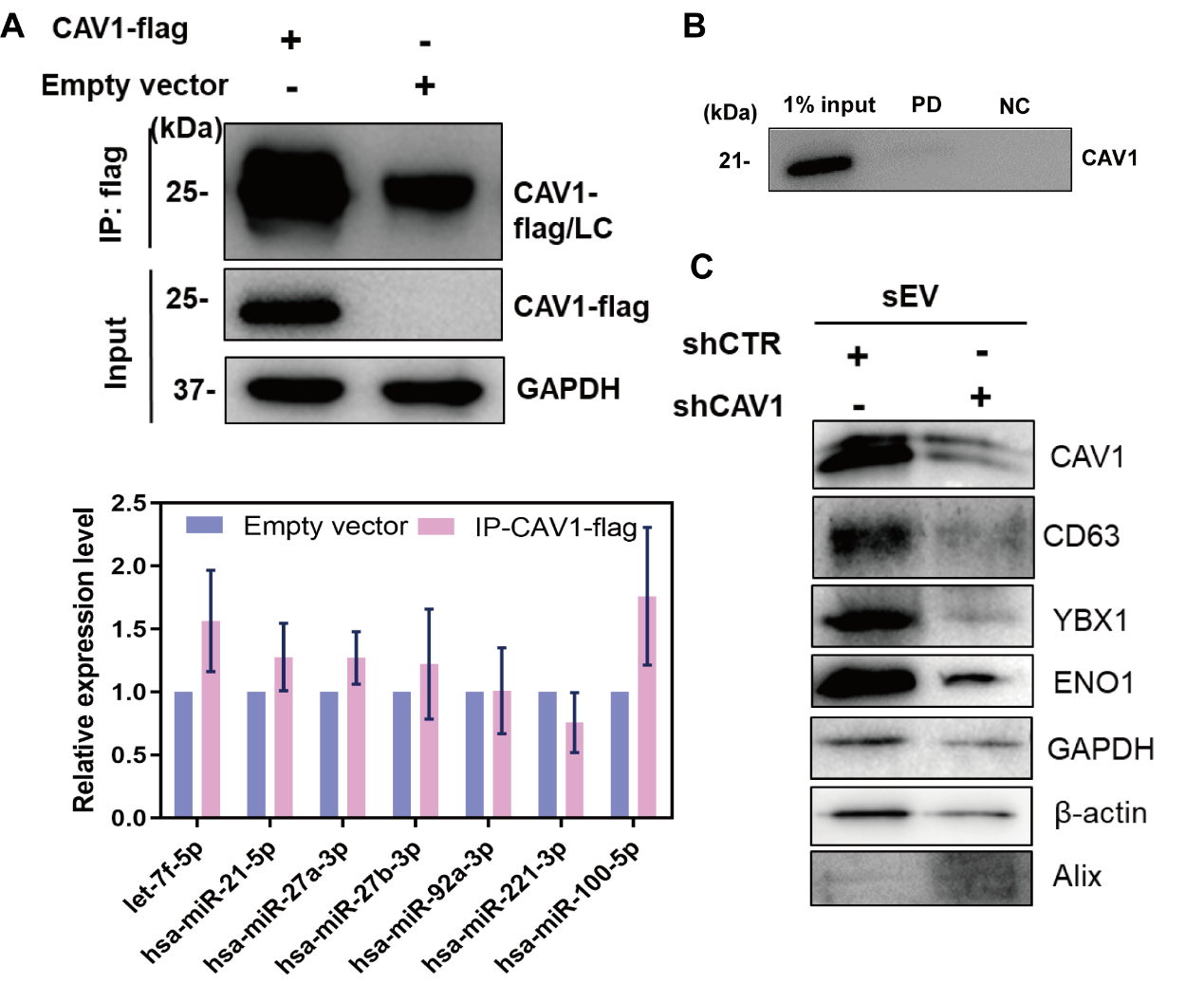


**Supplementary Figure 4.** (A) CAV1-flag fusion protein was purified in CAV1-flag overexpressed cell extracts (upper panel) and miRNAs in co-precipitated complex were detected by RT-qPCR (lower panel). Two independent assays were carried out. (B) CAV1 were detected in miR-221-3p binding complex. NC, negative control (beads + control miRNA without biotin). PD, pull-down (beads + biotin-labeled miR-221-3p). (C) Alteration of proteins (CAV1, CD63, YBX1, ENO1, GAPDH, β-actin and Alix) in sEV after knockdown of CAV1.

Supplementary tables

**Supplementary** **Table 1.** Primer sequences used in this study

| Gene Name | Primer Sequence (5'-3') | |
| --- | --- | --- |
|  | Forward | Reverse |
| hnRNPA1 | ACGAAACCAAGGTGGCTATG | GTGCTTGGCTGAGTTCACAA |
| PTBP1 | GTTGGGTCGGTTCCTGCTAT | CGTCAGATCCCCGCTTTGTA |
| GADPH | CAAGGTCATCCATGACAACTTTG | GTCCACCACCCTGTTGCTGTAG |
| hsa-mir-21-5p | ACACTCCAGCTGGG TAGCTTATCAGACTGA | universal reverse primer |
| hsa-let-7f-5p | ACACTCCAGCTGGG TGAGGTAGTAGATTG | universal reverse primer |
| hsa-mir-27a-3p | ACACTCCAGCTGGG TTCACAGTGGCTAAGTTCC | universal reverse primer |
| hsa-mir-27b-3p | ACACTCCAGCTGGG TTCACAGTGGCTAAGTTCT | universal reverse primer |
| hsa-mir-92a-3p | ACACTCCAGCTGGG TATTGCACTTGTCCCG | universal reverse primer |
| has-mir-221-3p | ACACTCCAGCTGGG AGCTACATTGTCTGCTG | universal reverse primer |
| hsa-mir-100-5p | ACACTCCAGCTGGGAACCCGTAGATCCGAA | universal reverse primer |
| has-let-7i-5p | ACACTCCAGCTGGG TGAGGTAGTAGTTTGTGC | universal reverse primer |
| hsa-mir-1246 | ACACTCCAGCTGGG AATGGATTTTTGG | universal reverse primer |
| hsa-mir-423-5p | ACACTCCAGCTGGG TGAGGGGCAGAGAGCGA | universal reverse primer |
| hsa-mir-320b | ACACTCCAGCTGGG AAAAGCTGGGTTGAGAGGGCA | universal reverse primer |
| cel-miR-39-3p | ACACTCCAGCTGGG TCACCGGGTGTAAATCAGCTTG | universal reverse primer |
| RNU6 | CTCGCTTCGGCAGCACA | universal reverse primer |

**Supplementary** **Table 2.** The miR-320b precipitated proteins detected by high through-put MS

| prot_acc | Gene Name | prot_score | prot_mass | prot_matches | prot_matches_sig | prot_sequences | prot_sequences_sig | prot_cover (%) |
| --- | --- | --- | --- | --- | --- | --- | --- | --- |
| P31943 | HNRNPH1 | 1191 | 49484 | 95 | 55 | 18 | 16 | 54.1 |
| P09651 | HNRNPA1 | 1187 | 38837 | 71 | 38 | 18 | 13 | 46.8 |
| P55795 | HNRNPH2 | 869 | 49517 | 81 | 44 | 15 | 12 | 39.6 |
| P52597 | HNRNPF | 828 | 45985 | 78 | 42 | 14 | 12 | 51.1 |
| Q9H3P7 | ACBD3 | 559 | 60841 | 58 | 23 | 13 | 7 | 47.9 |
| P09211 | GSTP1 | 391 | 23569 | 21 | 9 | 7 | 5 | 50.5 |
| P22626 | HNRNPA2B1 | 295 | 37464 | 33 | 16 | 14 | 9 | 46.7 |
| P04406 | GAPDH | 257 | 36201 | 18 | 11 | 9 | 7 | 40.3 |
| P00352 | ALDH1A1 | 253 | 55454 | 20 | 7 | 13 | 6 | 31.9 |
| P26599 | PTBP1 | 191 | 57357 | 6 | 5 | 4 | 3 | 11.5 |
| P06576 | ATP5B | 190 | 56525 | 12 | 7 | 9 | 4 | 29.3 |
| P30038 | ALDH4A1 | 179 | 62137 | 2 | 2 | 1 | 1 | 3.2 |
| P31942 | HNRNPH3 | 167 | 36960 | 15 | 9 | 10 | 7 | 30.9 |
| P06733 | ENO1 | 166 | 47481 | 20 | 8 | 13 | 6 | 42.2 |
| P16104 | H2AFX | 150 | 15135 | 4 | 3 | 2 | 1 | 25.2 |
| P0C0S8 | HIST1H2AG | 150 | 14083 | 5 | 4 | 3 | 2 | 42.3 |
| Q12849 | GRSF1 | 143 | 53606 | 17 | 7 | 10 | 6 | 30.8 |
| Q6FI13 | HIST2H2AA3 | 124 | 14087 | 6 | 4 | 3 | 2 | 42.3 |
| P10809 | HSPD1 | 115 | 61187 | 5 | 4 | 4 | 4 | 10.3 |
| P09104 | ENO2 | 114 | 47581 | 12 | 5 | 4 | 3 | 14.1 |
| P25705 | ATP5A1 | 114 | 59828 | 13 | 3 | 7 | 3 | 17.7 |
| P02462 | COL4A1 | 108 | 161654 | 3 | 3 | 2 | 2 | 2.4 |
| P31040 | SDHA | 101 | 73672 | 3 | 1 | 3 | 1 | 10.1 |
| P02768 | ALB | 97 | 71317 | 6 | 2 | 4 | 1 | 7.2 |
| P67809 | YBX1 | 91 | 35903 | 7 | 2 | 5 | 2 | 15.1 |
| P13929 | ENO3 | 90 | 47299 | 8 | 4 | 4 | 3 | 14.1 |
| Q5VTE0 | EEF1A1P5 | 86 | 50495 | 11 | 3 | 7 | 3 | 22.7 |
| P08263 | GSTA1 | 85 | 25672 | 1 | 1 | 1 | 1 | 12.2 |
| P13804 | ETFA | 84 | 35400 | 3 | 3 | 2 | 2 | 15 |
| Q02218 | OGDH | 72 | 117059 | 2 | 2 | 2 | 2 | 3.4 |
| Q562R1 | ACTBL2 | 63 | 42318 | 5 | 2 | 3 | 1 | 10.9 |
| Q15084 | PDIA6 | 60 | 48490 | 2 | 1 | 2 | 1 | 6.6 |
| Q8NAB2 | KBTBD3 | 57 | 70775 | 4 | 3 | 1 | 1 | 1.2 |
| P51991 | HNRNPA3 | 54 | 39799 | 8 | 5 | 2 | 2 | 7.1 |
| P30048 | PRDX3 | 54 | 28017 | 1 | 1 | 1 | 1 | 9.8 |
| Q13151 | HNRNPA0 | 48 | 30993 | 4 | 4 | 1 | 1 | 2.3 |
| Q96HY7 | DHTKD1 | 48 | 103752 | 4 | 1 | 2 | 1 | 6.1 |
| P05141 | SLC25A5 | 46 | 33059 | 2 | 1 | 2 | 1 | 9.4 |
| P12236 | SLC25A6 | 46 | 33073 | 2 | 1 | 2 | 1 | 9.4 |
| Q71DI3 | HIST2H3A | 46 | 15436 | 2 | 2 | 1 | 1 | 23.5 |
| Q92734 | TFG | 46 | 43478 | 5 | 2 | 4 | 2 | 18 |
| P30838 | ALDH3A1 | 42 | 50762 | 2 | 1 | 2 | 1 | 6.8 |
| P48735 | IDH2 | 42 | 51333 | 4 | 1 | 3 | 1 | 13.5 |
| Q3MJ13 | WDR72 | 38 | 125683 | 4 | 3 | 1 | 1 | 0.5 |
| Q15119 | PDK2 | 36 | 46352 | 1 | 1 | 1 | 1 | 8.1 |
| Q86U44 | METTL3 | 36 | 65175 | 3 | 1 | 1 | 1 | 1.4 |
| P05091 | ALDH2 | 36 | 56859 | 4 | 1 | 3 | 1 | 7.7 |
| P07954 | FH | 34 | 54773 | 1 | 1 | 1 | 1 | 8 |
| Q6UXN9 | WDR82 | 34 | 35456 | 2 | 2 | 1 | 1 | 1.9 |
| P14618 | PKM | 32 | 58470 | 7 | 1 | 6 | 1 | 16 |
| Q8TER0 | SNED1 | 32 | 158206 | 1 | 1 | 1 | 1 | 0.5 |
| P27144 | AK4 | 29 | 25366 | 1 | 1 | 1 | 1 | 5.4 |
| P26440 | IVD | 29 | 46803 | 2 | 1 | 1 | 1 | 5.9 |
| Q99943 | AGPAT1 | 28 | 32038 | 1 | 1 | 1 | 1 | 3.5 |
| Q6NXG1 | ESRP1 | 28 | 76449 | 2 | 2 | 1 | 1 | 0.9 |
| Q9BZC7 | ABCA2 | 28 | 272039 | 2 | 1 | 1 | 1 | 0.3 |
| P04004 | VTN | 28 | 55069 | 2 | 1 | 2 | 1 | 4.4 |
| O43150 | ASAP2 | 27 | 112835 | 4 | 1 | 1 | 1 | 0.8 |
| Q8NC51 | SERBP1 | 26 | 44995 | 1 | 1 | 1 | 1 | 3.9 |
| P11498 | PC | 26 | 130293 | 2 | 1 | 1 | 1 | 1.7 |
| Q13098 | GPS1 | 26 | 56071 | 1 | 1 | 1 | 1 | 1.2 |
| O75152 | ZC3H11A | 26 | 89931 | 1 | 1 | 1 | 1 | 0.7 |
| O60701 | UGDH | 26 | 55674 | 1 | 1 | 1 | 1 | 1.8 |
| P05455 | SSB | 26 | 46979 | 1 | 1 | 1 | 1 | 3.4 |
| P0C869 | PLA2G4B | 26 | 89006 | 2 | 1 | 1 | 1 | 1 |
| Q02252 | ALDH6A1 | 25 | 58259 | 2 | 1 | 1 | 1 | 5.4 |
| P49748 | ACADVL | 24 | 70745 | 1 | 1 | 1 | 1 | 2.6 |
| P0C0S5 | H2AFZ | 23 | 13545 | 2 | 1 | 2 | 1 | 28.1 |
| Q9HCD5 | NCOA5 | 21 | 65725 | 2 | 1 | 1 | 1 | 1.6 |
| P14923 | JUP | 19 | 82434 | 1 | 1 | 1 | 1 | 2.4 |
